# Geometric Assessment and Tissue Damage Control in Anatomically, Ultrasonographically, and Fluoroscopically Guided Intracapsular DICMO Osteotomies Conducted on Cadaveric Specimens

**DOI:** 10.3390/reports9010066

**Published:** 2026-02-19

**Authors:** Mario Suárez-Ortiz, María del Mar Ruiz-Herrera, Miguel López-Vigil, Eduardo Nieto-García, Sofía Mora-Pardo, Alfonso Martínez-Nova, Rodrigo Martínez-Quintana

**Affiliations:** 1 Clínica Podosalud, C/Sepúlveda 121, 28011 Madrid, Spain; footmario@hotmail.com (M.S.-O.); soooffmora@gmail.com (S.M.-P.); 2 Clínica Ruiz-Herrera, C/Trinidad 14, 13600 Alcázar de San Juan, Spain; clinicamariadelmarruiz@gmail.com; 3 Department of Nursing and Physiotherapy, Campus de Ponferrada, Universidad de León, 24401 León, Spain; mlopv@unileon.es; 4 Department of Podiatry, School of Medicine and Health Sciences, Universidad Católica de Valencia San Vicente Mártir, 46001 Valencia, Spain; eduardo.nieto@ucv.es; 5 Department of Nursing, University of Extremadura, 10600 Plasencia, Spain; 6 Department of Maths, University of Extremadura, 10600 Plasencia, Spain; rmartinez@unex.es

**Keywords:** metatarsal osteotomies, surgery, forefoot, metatarsalgia, joint capsule, geometry

## Abstract

**Introduction:** Distal intracapsular minimally invasive osteotomies (DICMOs) for central metatarsals are described as intracapsular procedures; however, neither their intracapsular location throughout the entire cut nor the optimal anatomical position for their execution have been fully validated. The aim of this study was to assess the geometric position of the DICMO osteotomy in the central metatarsals (third and fourth) and quantify associated anatomical damage when performed under three different guidance modalities: anatomical palpation, fluoroscopic control, and ultrasound guidance. **Material and methods**: An experimental cadaveric study was conducted using 29 fresh specimens (11 males, 18 females), contributing a total of 58 central metatarsals (third and fourth). All specimens underwent a DICMO-type metatarsal osteotomy. Osteotomies were randomly allocated to three intervention groups: (1) ultrasound (*n* = 20), (2) fluoroscopy (*n* = 19), and (3) anatomical guidance (*n* = 19). Metatarsal length, the distance between the osteotomy line and the articular surface, and post-dissection soft-tissue damage were recorded. **Results:** After dissection, all osteotomies were confirmed to be intracapsular. A constant proportional relationship was identified between osteotomy location and metatarsal length: distance to the joint line = 0.239 × metatarsal length. This relationship was independent of the guidance technique used. Only one iatrogenic lesion was observed: an articular cartilage injury of a third metatarsal in the anatomical-guidance group. **Conclusions:** The optimal position for DICMO osteotomy placement is approximately 24% of the total distal metatarsal length. This ensures an intracapsular trajectory and may contribute to intrinsic osteotomy stability. Image guidance—either fluoroscopy or ultrasound—appears essential to optimize outcomes and prevent avoidable anatomical damage.

## 1. Introduction

Surgical management of central-ray metatarsalgia (corresponding to the second–fourth metatarsophalangeal joints) remains challenging when conservative treatment fails [1]. Anatomically, the second and third metatarsals exhibit lower mobility than the fourth and fifth metatarsals, resulting in reduced compensatory motion at the tarsometatarsal joints. This is explained by their articulation with the second and third cuneiform bones, respectively, and their stabilization by the Lisfranc ligament, whereas the fourth and fifth metatarsals articulate with the cuboid, conferring greater mobility.

Minimally invasive (MIS) approaches rely on osteotomies conducted at the surgical neck of the affected metatarsal to achieve controlled shortening and elevation of the metatarsal head, thereby reducing plantar pressure caused by plantarflexed alignment. For mechanical metatarsalgia, distal minimally invasive metatarsal osteotomies (DMMOs) are frequently employed. These extra-articular distal osteotomies do not require fixation and have been proposed as an alternative to the traditional Weil osteotomy [2] due to potential advantages in dynamic correction, cost reduction (by avoiding osteosynthesis hardware) [3,4,5,6,7], and reduced joint stiffness [8]. DMMOs are technically simple, effective, and highly reproducible [8,9]. They are performed at the distal metaphyseal–diaphyseal transition, with an approximately 45° inclination relative to the metatarsal axis in the sagittal plane. With experience, the plane can be adjusted to optimize elevation or shortening [10].

However, DMMOs may result in undesired sequelae, including metatarsal head displacement, exuberant callus formation, prolonged oedema, floating toes, and transfer metatarsalgia. As a refinement, distal intracapsular minimally invasive osteotomies (DICMOs) were developed. These are more distal than DMMOs and fully intracapsular [11,12], increasing intrinsic stability and reducing the likelihood of metatarsal head displacement [12,13,14]. Their more distal location results in less shortening, thereby theoretically reducing overload transfer to adjacent rays.

Most DICMO procedures are performed under fluoroscopic control. Despite the low radiation output of modern mini C-arm systems, cumulative exposure for surgeons, particularly with respect to the eyes and hands [15], remains a concern. In some cases, osteotomies are performed using only anatomical landmarks; however, this approach lacks precision, as anatomical variability may lead to technical inaccuracies. In recent years, ultrasound-guided foot surgery has expanded, allowing direct identification of key intraoperative structures (joint capsule, cartilage, and tendons) while eliminating radiation exposure [16]. Ultrasound may enhance safety, particularly in MIS procedures where the visual field is limited.

We hypothesized that radiation exposure during DICMO could be avoided by using intraoperative ultrasound, without compromising accuracy. Furthermore, establishing a reliable geometric reference point for DICMO positioning could improve surgical planning, particularly during the learning curve. The objective of this study, therefore, was to determine the geometric location of the DICMO osteotomy in central metatarsals (third and fourth) and assess associated anatomical damage using anatomical palpation, fluoroscopic control, or real-time intraoperative ultrasonography.

## 2. Materials and Methods

### 2.1. Study Design

An experimental cadaveric study was performed on 29 fresh human cadaver specimens (11 males, 18 females), contributing 58 central metatarsals (3rd and 4th). Thirteen specimens were right feet, and sixteen were left feet. Osteotomies were randomized (using Research Randomizer software) into three groups: (1) ultrasound (*n* = 20), (2) fluoroscopy (*n* = 19), and (3) anatomical guidance (*n* = 19).

### 2.2. Surgical Technique

**Figure 1:**
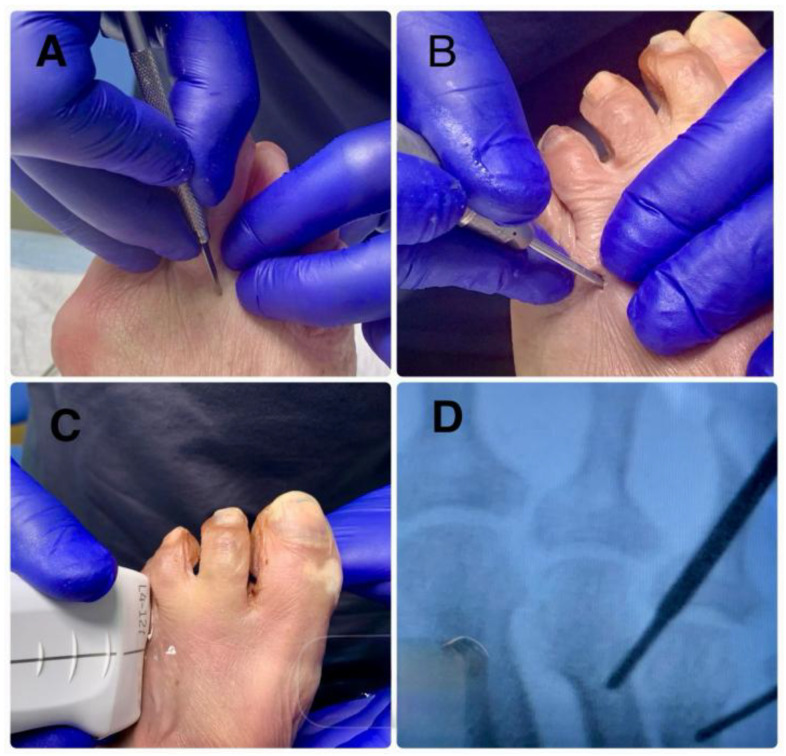
(**A**): Palpatory identification of the target zone for the surgical access portal. (**B**): Incision made with a Beaver 64 blade, advancing into the joint capsule at a 45° angle. (**C**): Ultrasonographic examination of the area where the subsequent incision and osteotomy were performed. (**D**): Fluoroscopic confirmation of the position of the long Shannon–Isham burr.

**Figure 2:**
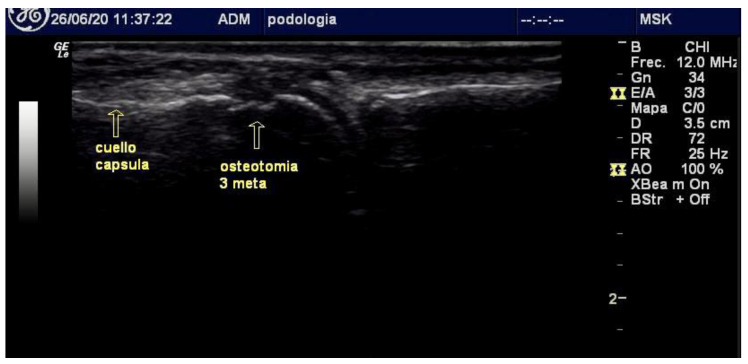
Ultrasound image showing how the intracapsular position of the osteotomy was evaluated. Considering that the joint capsule was inserted along the margins of the articular surfaces of the metatarsal head and the base of the corresponding phalanx, and that the articular cartilage is thicker on the plantar aspect of the metatarsal head, this enables accurate placement of the osteotomy within this region at the desired geometric location, under direct fluoroscopic and ultrasound guidance, using these anatomical landmarks.

1. The metatarsophalangeal joint and metatarsal head were palpated using the non-dominant hand. Dorsally, the index and middle fingers were used to assess joint flexion–extension; plantarly, the thumb was used to stabilize the metatarsal head to prevent displacement.
2. A dorsal incision perpendicular to the skin was made slightly medially or laterally, depending on foot laterality, to facilitate burr rotation. The incision was made distal to the metatarsal head, allowing the osteotomy to proceed from distal–dorsal to proximal–plantar, parallel to the extensor tendons. The Beaver 64 blade was angled at 45° until the dorsal–distal capsule entered precisely at the origin of the articular cartilage, avoiding cartilage violation.
3. The Beaver 64 blade was advanced at a 45° angle relative to the metatarsal axis toward the periosteum of the metatarsal neck.
4. Within the intracapsular plane, the blade (or, alternatively, a rasp) was used to elevate the periosteum and lightly score the cortical surface to prevent burr skiving during initial bone contact.
5. A straight-fluted Shannon-Isham burr was inserted along the incision trajectory, placed into the previously created cortical notch, and oriented at 45° to the metatarsal axis. With gentle distal advancement, the surgeon palpated the metatarsal head condyle as a mechanical stop before completing the intracapsular minimally invasive osteotomy, or DICMO [17].
6. In the anatomical-guidance group, burr trajectory relied solely on palpatory landmarks. In the fluoroscopy group, a portable mini C-arm unit (Fluoroscan Insight 2, Hologic, CA, USA) was used under strict radiation-safety protocols. In the ultrasound group, burr advancement and visualization of adjacent structures were monitored in real-time with a high-frequency linear probe (GE LOGIQ R8, General Electric, IL, USA). Once alignment was confirmed, a long Shannon–Isham burr was used at 250 rpm to perform the osteotomy from the distal–dorsal to proximal–plantar directions (Figure 1 and Figure 2).

### 2.3. Radiological Variables

**Figure 3:**
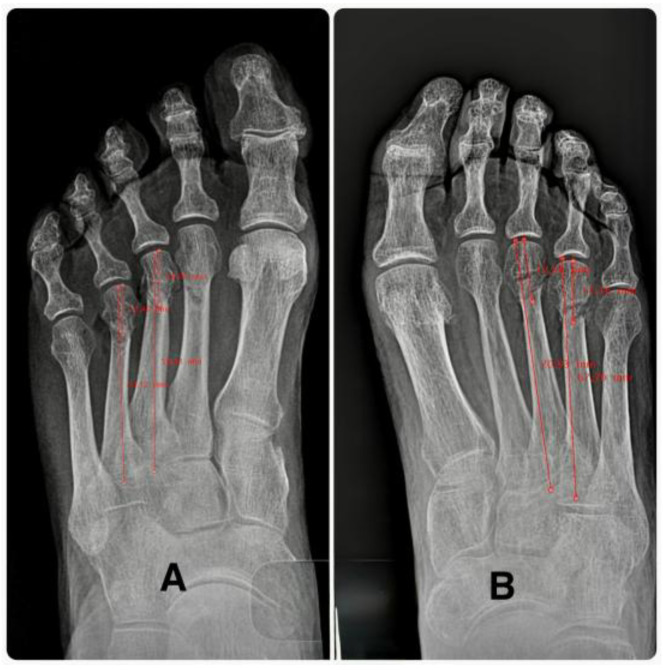
(**A**): Xray measurements before the osteotomy. Length of 3rd and 4th metatarsals (mm) (**B**). Osteotomy measurement. Distance from the osteotomy to the articular line (mm).

A standardized dorsoplantar radiograph (1 m distance, 60 kV, 2 ms) was used to determine (1) metatarsal length, in mm, and (2) the distance from osteotomy to the articular line, in mm (Figure 3). Author 4, who was blinded with respect to group assignment, used radiographic workstation software to make measurements.

### 2.4. Dissection of Specimens and Anatomical Tissue Damage Variables

Two experts in foot surgery and dissection (authors two and three), who were not present when the techniques were performed and thus blinded with respect to group assignment, evaluated the anatomical damage caused by the three technical modalities by dissecting the specimens. Through a dorsal incision in the joint, they examined the dorsal structures, extensor tendons, blood vessels, and nerves, without finding any relevant damage. Subsequently, the incision was deepened until the capsule was reached, and the experts evaluated the position of the osteotomies, i.e., whether or not they were inside the articular capsule. Upon opening the capsule, the metatarsal heads were removed to evaluate tissue damage. The dissectors also evaluated some categorical variables (yes/no): a—capsular damage (when the opening of the capsule was more than 1 mm wider than the width of the scalpel, i.e., the Beaver 64); b—plantar plate injury (when the plantar plate showed superficial abrasions/erosions at the osteotomy site); c—contact between the burr and the extensor tendons, extensor digitorum longus, or extensor digitorum brevis; and d—contact between the burr and the joint capsule (when the tendons or capsule showed abrasions/erosions due to the passage of the Isham burr).

### 2.5. Statistical Analysis

Descriptive statistics included absolute (N) and relative (%) frequencies, means, and standard deviations. Kruskal–Wallis testing confirmed group homogeneity regarding metatarsal length (*p* = 0.101). Sex distribution (Table 1, *p* = 0.665) and foot laterality (*p* = 0.142) were also homogeneous.

A no-intercept linear regression model was used to examine the relationship between total metatarsal length and osteotomy distance. Statistical analyses were conducted using SPSS v29 (IBM, Armonk, NY, USA; UEX Campus license).

## 3. Results

### 3.1. Anatomical Damage

**Figure 4:**
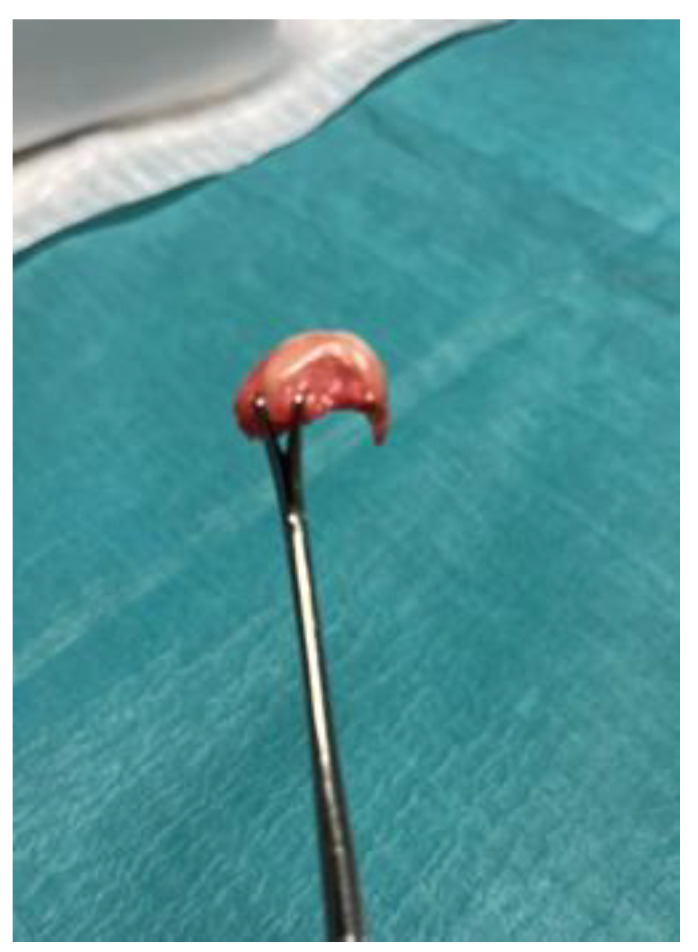
Dissection of the metatarsal head showing articular cartilage damage in the anatomical-guidance technique caused by performing an excessively distal osteotomy.

**Figure 5:**
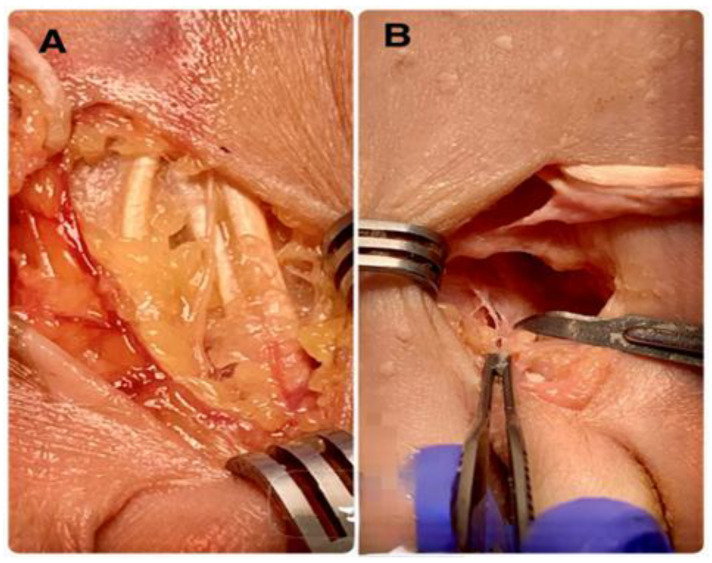
(**A**): Dissection of the dorsal structures demonstrating their integrity. (**B**): Confirmation of the absence of injuries of the plantar structures and the flexor plate.

The dissection revealed a lesion of the articular cartilage in the third metatarsal of the anatomical guide group caused by an excessively distal osteotomy in the plantar area (Figure 4). The final result was that all osteotomies were intracapsular and had a dorsal–distal to plantar–proximal orientation, as expected. No additional soft tissue injuries occurred beyond the surgical portal. Neither the plantar plate nor the extensor or flexor tendons were compromised (Figure 5). All results were considered adequate except for damage to the articular cartilage produced in the anatomical guidance modality.

### 3.2. Osteotomy Geometry

Metatarsal length was significantly greater in males (*p* < 0.001). No significant differences were found in osteotomy-to-joint-line distance between groups (*p* = 0.488). A highly significant regression model (*p* < 0.001, R^2^ = 0.975) was obtained: D = 0.239 × L. This model was independent of guidance modality (*p* = 0.711) and foot laterality (*p* = 0.416). The sex-specific coefficients differed slightly (women: D = 0.247 × L; men: D = 0.228×L), reflecting differences in metatarsal length.

## 4. Discussion

Our findings demonstrate strong reproducibility of the osteotomy cutting angle, indicating that fluoroscopic guidance combined with ultrasound imaging and anatomical palpation is feasible and reliable in a cadaveric setting. Moreover, the distance from the intracapsular osteotomy to the joint line proved to be essentially constant and independent of the imaging guidance method used. This supports the notion that DICMO osteotomies, when performed at an appropriate proportional distance relative to the total metatarsal length, consistently remain intracapsular.

To date, no previous studies have quantified the precise geometric location at which a DICMO osteotomy should be performed on the lesser metatarsals. In this study, we provide new evidence that defines a reproducible proportional reference point: approximately 23–24% of the total length of the metatarsal when measured distally. Our establishment of this geometric reference may facilitate preoperative planning, improve consistency of technique among surgeons with varying levels of experience, and potentially shorten the learning curve in minimally invasive forefoot surgery.

In terms of safety, only one sample showed iatrogenic cartilage damage, and this occurred exclusively in the anatomical guidance group, without any imaging support. We have found that this reinforces the importance of using real-time visualization, whether fluoroscopic or ultrasound, to avoid conducting an osteotomy too distally and protect adjacent structures. It is important to note that no injuries were identified in the plantar plate, dorsal extensor tendons, flexor apparatus, or periarticular capsule in any of the three groups [12,16,18]. These findings suggest that DICMO can be performed safely within the joint capsule, with minimal disruption of collateral soft tissues when properly guided.

Ultrasound-guided techniques are increasingly integrated into foot and ankle surgery due to their capacity to visualize soft-tissue structures, avoid radiation exposure [15], and offer real-time dynamic assessment. In one study, the authors found that ultrasound guidance reliably confirmed intracapsular positioning and allowed visualization of the osteotomy trajectory in relation to the capsule, cartilage, and tendons [16]. This may be of particular relevance for surgeons aiming to limit radiation exposure, especially considering the cumulative occupational risks to the eyes and hands, which are well documented in fluoroscopy-dependent procedures. However, despite the favorable clinical outcomes that ultrasonography may offer, its routine use presents several practical limitations. From a clinical point of view, ultrasound-guided assessment is more time-consuming than conventional radiographic methods and requires specific training, being inherently operator-dependent. Distal metatarsal osteotomies are commonly performed in minimally invasive surgery (MIS) settings, where fluoroscopic guidance with a C-arm is routinely used throughout the procedure. In this context, ultrasound guidance could be a viable alternative when these procedures are performed in isolation. Nevertheless, in combined surgical interventions, such as those addressing hallux valgus, we believe that the integration of an additional imaging modality may disrupt surgical workflow and operative efficiency, as it necessitates the incorporation of extra equipment into an already complex operating theatre setup.

We established a proportional model (D = 0.239 × L) that demonstrated excellent predictive capacity (R^2^ = 0.975), suggesting that metatarsal length is a robust parameter for anticipating the ideal osteotomy site. Sex-based differences (D = 0.247 × L for females; D = 0.228 × L for males) appear to reflect anatomical variation in metatarsal dimensions rather than meaningful changes in the required surgical landmark. Therefore, using a proportional reference rather than an absolute distance will ensure the method’s applicability across different anatomies.

This geometric consistency supports biomechanical hypotheses that more distal intracapsular osteotomies may enhance intrinsic stability by maintaining the osteotomy within the capsular envelope and reducing the risk of uncontrolled displacement of the metatarsal head. Furthermore, because DICMO techniques result in less shortening compared with extra-articular DMMO osteotomies, the risk of symptomatic load transfer to adjacent rays may theoretically be reduced.

The limitations of the present study include the use of cadaveric specimens, which cannot fully replicate in vivo soft-tissue tension, vascularity, or dynamic biomechanical forces. Additionally, the number of specimens, although adequate for a controlled anatomical study, may have limited subgroup analyses. Future clinical research should investigate whether these geometric principles correlate with improved clinical outcomes, reduced complication rates, and enhanced postoperative stability in live patients.

## 5. Conclusions

The optimal geometric location for a DICMO intracapsular osteotomy is approximately 24% of the total distal metatarsal length, and the surgeon must ensure that the cut remains reliably within the joint capsule. This proportional reference appears independent of imaging modality, foot laterality, or specimen group. Image guidance, particularly fluoroscopic or ultrasonographic, is strongly recommended to prevent inadvertent cartilage injury and optimize the precision and safety of the procedure. Ultrasound, in particular, offers a radiation-free alternative allowing clear visualization of periarticular structures. These findings provide a reproducible anatomical and geometric framework for improving accuracy and safety in minimally invasive intracapsular metatarsal osteotomies.

## Figures and Tables

**Table 1:** Osteotomy distribution by group and gender (chi-squared test).

	Group			Overall
	Ultrasound	Fluoroscopy	Anatomy	
Men	6 30%	8 42.1%	8 42.1%	22 37.9%
Women	14 70%	11 57.9%	11 57.9%	36 62.1%
χ^2^	0.665			

## Data Availability

The original contributions presented in the study are included in the article, further inquiries can be directed to the corresponding author.
